# Excessive Intrauterine Fluid Cause Aberrant Implantation and Pregnancy Outcome in Mice

**DOI:** 10.1371/journal.pone.0078446

**Published:** 2013-10-23

**Authors:** Shan Lu, Hongying Peng, He Zhang, Li Zhang, Qichen Cao, Rong Li, Ying Zhang, Liying Yan, Enkui Duan, Jie Qiao

**Affiliations:** 1 Department of Obstetrics and Gynecology, Peking University Third Hospital, Beijing, China; 2 State Key Laboratory of Reproductive Biology, Institute of Zoology, Chinese Academy of Sciences, Beijing, China; 3 Beijing Key Laboratory of Reproductive Endocrinology and Assisted Reproductive Technology, Beijing, China; 4 Key Laboratory of Assisted Reproduction, Ministry of Education, Beijing, The People’s Republic of China; The University of Georgia, United States of America

## Abstract

The normal intrauterine fluid environment is essential for embryo implantation. In hydrosalpinx patients, the implantation and pregnancy rates are markedly decreased after IVF–embryo transfer, while salpingectomy could significantly improve the pregnancy rates. The leakage of hydrosalpinx fluid into the endometrial cavity was supposed to be the major cause for impaired fertility. However, the underlying mechanisms of hydrosalpinx fluids on implantation and ongoing pregnancy were not fully understood and remain controversial regarding its toxicity. In present study, by infusing different volume of non-toxic fluid (0.9% saline) into uterine lumen before embryo implantation in mice (Day4 08:30), we found that while the embryos were not “flushed out” from the uteri, the timing of implantation was deferred and normal intrauterine distribution (embryo spacing) was disrupted. The abnormal implantation at early pregnancy further lead to embryo growth retardation, miscarriage and increased pregnancy loss, which is similar to the adverse effects observed in hydrosalpinx patients undergoing IVF-ET. We further examined uterine receptivity related gene expression reported to be involved in human hydrosalpinx (*Lif, Hoxa10, Integrin α(v*)* and β(3*)). The results showed that expression of *integrin* α(*v*) *and* β(3) were increased in the fluid infused mouse uteri, implicating a compensatory effect to cope with the excessive fluid environment. Our data suggested that the adverse effects of excessive non-toxic luminal fluid on pregnancy are primarily due to the mechanical interference for normal timing and location of embryo apposition, which might be the major cause of decreased implantation rate in IVF-ET patients with hydrosalpinx.

## Introduction

The mammalian embryo implantation happens in a receptive endometrium with well-regulated fluid environments [1]. When the embryo(s) enter the uteri, intraluminal fluid provided the buffer to carry the embryos and facilitate their transport to the correct intrauterine location [1]. Before the embryo(s) attached to the uterine wall, on-time reabsorption of intra-luminal uterine fluid is supposed to be necessary for uterine luminal closure, which facilitates the interactions between the embryo and the epithelial lining to initiate attachment reaction [1,2]. This assumption was supported by the observation that uterine luminal fluid reabsorption peaks at the expected time of implantation in rodents [3,4], which is controlled by concerted ovarian hormone secretions and local ion and water channels [1,3–6]. 

The importance of uterine fluid control for embryo implantation is also well recognized in clinical protocols of embryo transfer (ET) after IVF. During the procedure of embryo transfer, only limited volume of fluid (usually 20-60 µL) was allowed to be co-transferred with embryo(s), as to preclude the extensive floating of the transferred embryo(s) [7]. Excessive uterine fluid at the time of implantation could lead to infertility. For example, in women with hydrosalpinx (with blocked and fluid filled fallopian tube), the implantation rates under IVF-ET cycles were significantly reduced, and the miscarriage rates approximately doubled during mid-pregnancy [8,9]. The leakage of hydrosalpinx fluid into the endometrial cavity was supposed to be the major cause for the low IVF success rate [10–12]. This idea has been substantiated by the facts that treatments such as salpingectomy/occlusion of proximal tube, which prevent the leakage of hydrosalpinx fluid, could significantly improve the success of IVF-ET in treated patients [13–16].

Currently existing hypotheses concerning the adverse effects of excessive fluid could be cataloged in several major aspects: 1) a direct toxic effect on developing embryo due to the inflammatory nature. 2) Mechanically interfere with embryonic apposition. 3) “Flush out” of embryos. 4) altered endometrial receptivity with changed gene expression [17–19]. However, the relative significance of these factors remained unclear and controversial. In present study, we developed an animal model by intraluminal infusion of non-toxic fluid (saline) at mice preimplantation, mimicking the leakage of excessive hydrosalpinx fluid into uterine cavity, and track the state of embryo implantation and ongoing pregnancy. We found that the excessive non-toxic intraluminal fluid before implantation indeed decreased the number of embryo implantation and increased miscarriage rate in a dose dependent manner, indicating that the inflammatory nature of fluid is not necessary for the adverse effects. Also, the major cause of excessive fluid in our study is not due to the “flush out” of embryos, but because of delayed timing and aberrant embryo spacing at embryo implantation. Some epithelial biomarkers for embryo-epithelium attachments also showed changed expression. Our data using mouse model provided coherent explanations to hydrosalpinx’s adverse effects on IVF-ET outcome, and also raised the caution to use intraluminal injection as a method to study embryo implantation factors.

## Materials and Methods

### Ethics Statement

The Guidelines for the Care and Use of Animals in Research were followed. Mice care and handling were conducted in accordance with the Animal Research Committee guidelines of the Institute of Zoology, Chinese Academy of Sciences. The institute does not issue a number for each animal study, but there is an ethical committee to guide animal use. The contents in present study regarding animal uses were approved by the Animal Research Committee of the Institute of Zoology, Chinese Academy of Sciences. 

### Animals and interventions

CD1 female mice (7-8 weeks) used in this study were purchased from Vital River Laboratories Co. Ltd. All mice were fed in the animal facility of Institute of Zoology, Chinese Academy of Sciences. The mice were maintained in 12-h light, 12-h dark conditions and given water and food freely. Adult female mice were mated with fertile males of CD1 at room temperature (25 °C). The morning of finding a vaginal plug was designated as Day1 of pregnancy. Intrauterine saline (0.9 %) infusion was performed on Day4 08:30, after the mice were under anesthesia by ip injection of avertin. Both uterine horns were infused with same volume of saline (0, 3, 10 and 20 μl) from the proximal end early the oviduct (using a needle gently pricking into the uterine lumen). The saline used in this study was for routine medical use in Peking University Third Hospital, and were further sterilized by autoclave before intrauterine infusion. The 0 μl group means that the mice were under the same surgical procedure but only with an empty needle pricking into the uterine lumen without fluid infusion. The intrauterine infusion processes were performed steady and slow, by using fine needles with measuring range 10 μl or 20 μl, as to minimize the possible fluid current that flush the embryos away.

### Examination of intrauterine embryos

After intrauterine saline fusion on Day4 08:30, the mice were sacrificed on Day4 16:00 or 21:00 and their uteri were flushed with saline solution, the intrauterine embryo were collected, counted and photographed under microscope. 

### Examination of implantation sites

The implantation sites on Day5 and Day6 were identified by intravenous injection of 0.1 ml of 1 % Chicago blue dye (Sigma) in saline as previously described [20]. Uteri without visible blue band on Day5 were further flushed to examine whether there are unimplanted blastocysts. 

### Examination of mid-gestation and litter size

Mice were sacrificed on Day12 of pregnancy to examine the mid-gestation status after intrauterine saline infusion (0, 10 and 20 μl) on Day4. Resorption sites were recorded; some embryos were isolated to examine the developmental status. Litter sizes for each group were calculated on the day of parturition. 

### RNA extraction, reverse transcription and quantitative PCR

RNA extraction and reverse transcription were performed as previously described [21]. In brief, total RNA was extracted from fresh tissues using the TRIzol reagent (Invitrogen) according to the manufacturer’s protocol, and genomic DNA was removed using the RNase-free DNase (Promega). After reverse transcription, SYBR Green-based quantitative RT-PCR was carried out using LightCycler 480 II (Roche). Primers for *Lif, Hoxa10, Muc1, Integrin α*(*v*) *and* β(3) are listed below: *Lif*-forward: 5’-ATTGTGCCCTTACTGCTGCTG-3’, *Lif*-reverse: 5'-GCCAGTTGATTCTTGATCTGGT-3'; *Hoxa10*-forward: 5'-ACGATGCTGCGGACAAAT-3', *Hoxa10*-reverse: 5'-TGCGACAGGCGGAAGTAG-3'; *Muc1*-forward: 5'-GCTGGTGCTGGTCTGTAT-3', *Muc1*-reverse: 5'-CGTAGCGTCCGTGAGTGT-3'; *Integrin* α(*v*)-forward: 5'-CCCAAAGCGAACACGACC-3', *Integrin* α(*v*)-reverse: 5'-CACAGAGGCTCCAAACCA-3'; *Integrin* β(3)-forward: 5'-CCCCGATGTAACCTGAAGGAG-3', *Integrin* β(3)-reverse: 5'-GAAGGGCAATCCTCTGAGGG-3'.

### Statistical analysis

Statistical analyses were performed with SPSS 17.0. All data are present as mean ± S.E.M of at least three independent experiments. Results were analyzed by one-way ANOVA or independent *t* test. *P* < 0.05 was considered to be statistically significant.

## Results

### Intrauterine infusion of saline at preimplantation cause decreased implantation rates in a dose dependent manner

**Figure 1:**
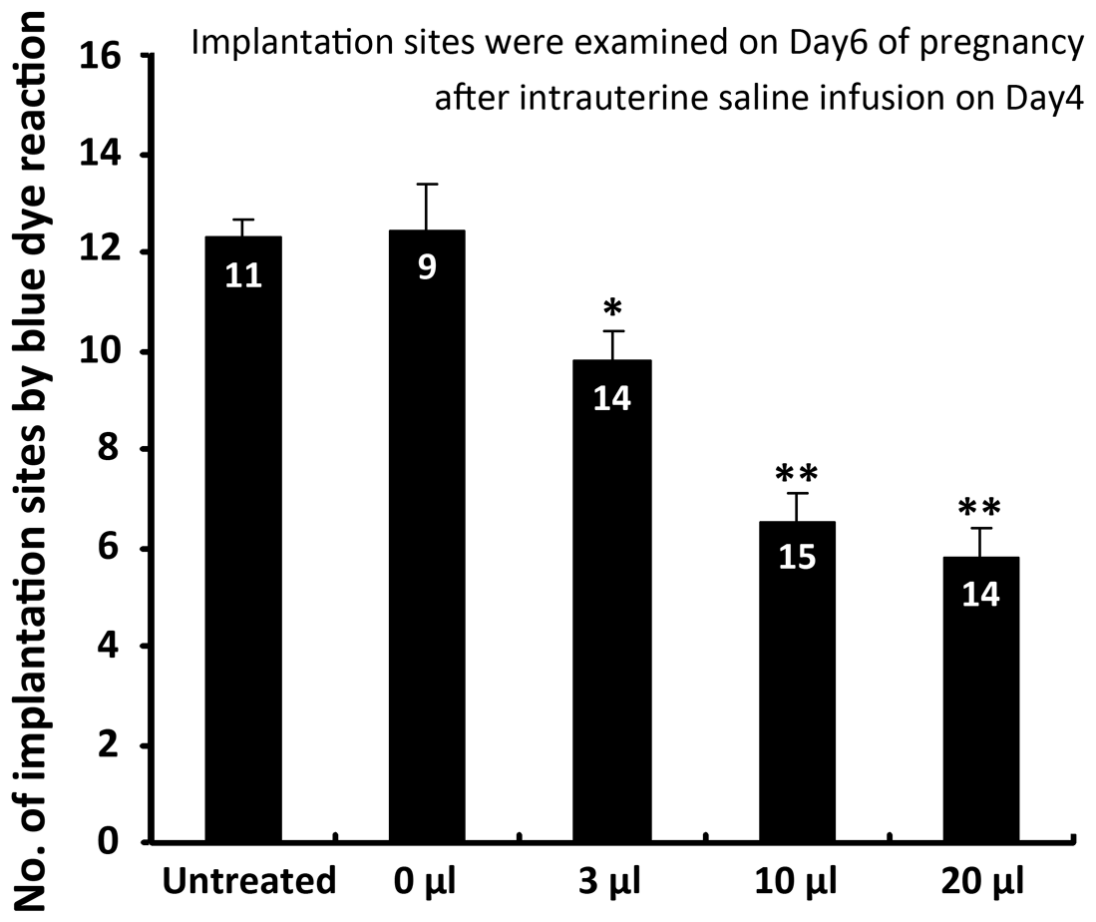
Intrauterine infusion of excessive non-toxic fluid (saline) at preimplantation caused decreased implantation sites in mice. Different volume of intrauterine fluid infusion (0 μl, 3 μl, 10μl and 20 μl) were performed on Day4 (08:30), the number of implantation sites were examined on Day6 (09:00) using blue dye reaction. Numbers of implantation were compared with untreated group. (*P < 0.05, **P < 0.01, *t* test, Error bars represent S.E.M) Numbers in the bars mean number of mice used in each group.

To examine the hypothesis that whether the previously supposed toxic components of hydrosalpinx fluid is necessary to cause adverse embryo implantation, here we firstly used the sterilized saline for intrauterine infusion at preimplantation uteri (at Day4 08:30), and examined the implantation rate at Day6 by blue dye reaction. As shown in Figure 1, even a small volume of intrauterine saline infusion (3 μl) could cause significant decrease of implantation sites, and the adverse effects were more substantial by 10 μl and 20 μl infusion. The 0 μl group means that the mice were under the same surgical procedure but only with an empty needle pricking into the uterine lumen without fluid infusion. These data clearly demonstrated that excessive intrauterine fluid, even by the non-toxic sterilized saline, could significantly decrease implantation rate, indicating that the inflammatory factors in fluid is not necessary for the adverse effects. 

### The decreased implantation rate was not due to “flush out” of embryos

**Figure 2:**
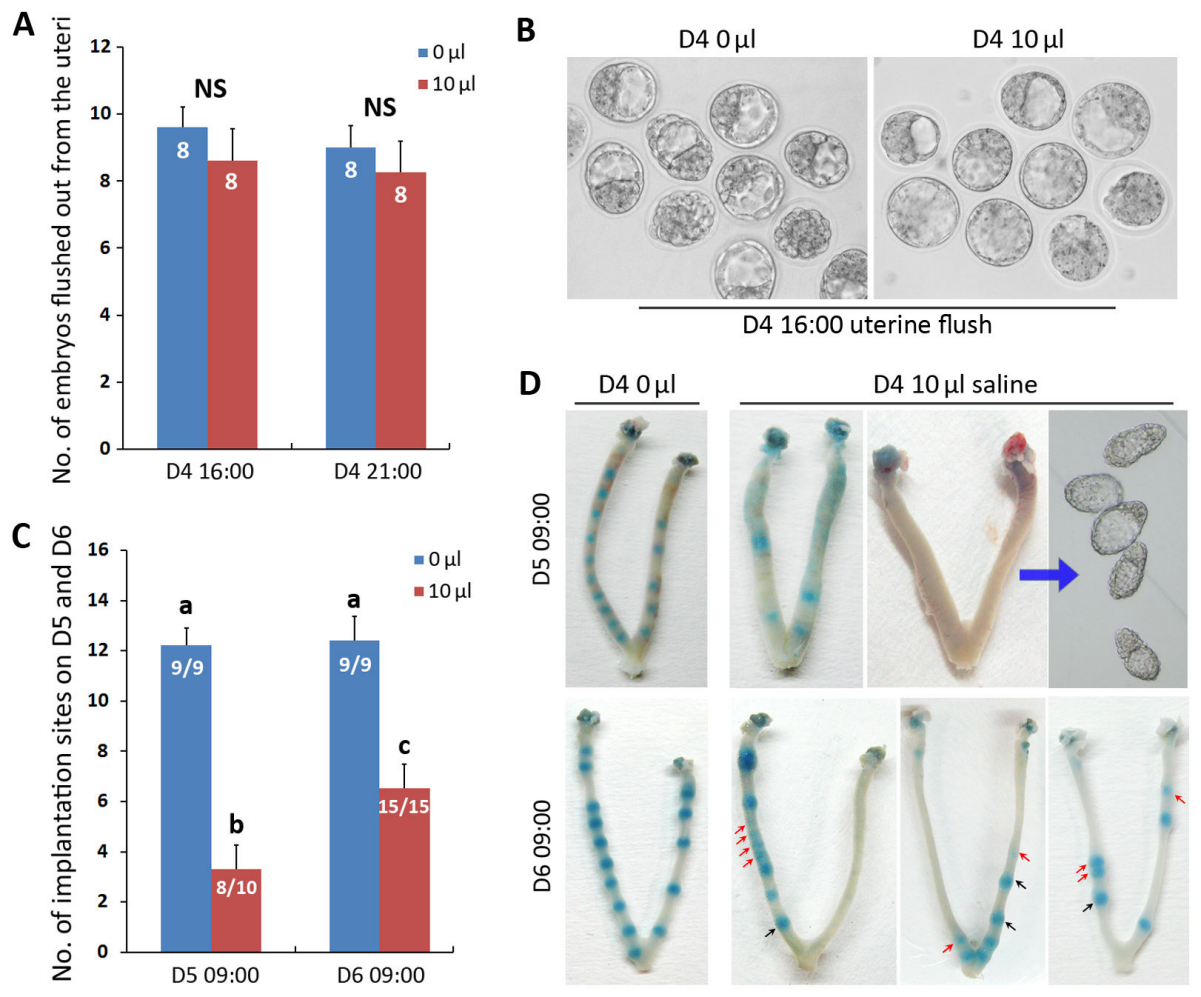
Cause of decreased implantation sites were not due to “flush out” of embryos, but due to abnormal “on time” and “on site” implantation. (**A**) The intrauterine embryo numbers were comparable between 10 μl and 0 μl fluid infused groups at different time points (NS: P > 0.05). (**B**) Demonstrative pictures of blastocysts flushed out at Day4 16:00 showed similar morphology between 10 μl and 0 μl fluid infused groups. (**C**) Number of implantation sites examined on Day5 and Day6 revealed delayed timing of implantation after 10 μl fluid infusion, groups with different superscript letters (a, b, c) are significantly different from each other (*P* < 0.01). Numbers within bars mean No. of mice with implantation sites/No. of mice examined. (**D**) Representative photos of normal and abnormal implantation sites examined on Day5 and Day6. Uteri without blue band were further processed for uterine flushing to examine whether blastocysts were existed. Note that embryo spacing was also disrupted especially on Day6. The black arrow showed relatively normal implantation sites, the red arrow showed delayed or abnormally spaced implantation sites.

We next examined the hypothesis whether the decreased implantation rate is due to excessive intrauterine fluid flush out the embryos from the uterus. By examining the embryos in the uteri at Day4 16:00 and 21:00, we found that the numbers of embryos in the uteri were similar in both 0 μl and 10 μl saline infusion (on Day4 08:30) groups (Figure 2A), indicating that most embryos still stay in the uterine lumen. Also, the morphology of the embryos after fluid infusion seems normal as compared with the 0 μl group (Figure 2B).

### The decreased implantation rate was due to delayed timing of embryo implantation with abnormal embryo spacing, associating with delayed luminal closure

However, when we examined the implantation sites by blue dye reaction on Day5 morning (09:00), we found implantation rate was significantly decreased in 10 μl saline infusion group, as compared with the 0 μl group (Figure 2C,D). After flushing the uteri with no observable implantation sites, we found that the unimplanted embryos were still in the uterine lumen with normal morphologies. For the 10 μl infusion group, implantation number was increased on Day6 morning (09:00) compared with Day5 (Figure 2C). The different size of implantation sites in the same uterus marked by blue band indicated some embryos showed delayed implantation (Figure 2D). These results demonstrated that the 10 μl intrauterine saline infusion at Day4 disrupted the timing of normal implantation. Besides the aberrant timing of embryo implantation, it is also notable that in 10 μl group, the normal embryo spacing is also disrupted in some uteri as showed in Figure 2D. 

Since successful embryo implantation requires an intimate interaction between the blastocyst and the luminal epithelia, which is facilitated by an important process called “luminal closure” [1,2,22]. We hypothesized that the delayed implantation and abnormal embryo spacing might be due to delayed luminal closure caused by excessive intrauterine fluid. By performing longitudinal uterine sections on flash frozen Day4 uteri (the frozen section ensured that the state of luminal closure will not be influenced by fixative agents or tissue dehydration), we found that by the time of 16:00 Day4, although the normal uteri have not undergo complete luminal closure, there are some luminal region has become closely opposed to each other (Fig. S1A), while the 10 μl saline infusion group showed no sign of luminal closure with clear gaps between the opposing epithelia (Fig. S1A). By the time of 24:00 Day4, luminal closure have been completed in control uteri(Fig. S1B), while in 10μl saline infusion uteri, there are still gaps remain between the opposing epithelia, although the width of the gap have been reduced (Fig. S1B). These data supported the hypothesis that the excessive luminal fluid indeed interfere the normal process of luminal closure that essential for the initiation of embryo implantation. 

### The abnormal timing and spacing at embryo implantation cause retarded embryo growth and miscarriage at mid-pregnancy

**Figure 3:**
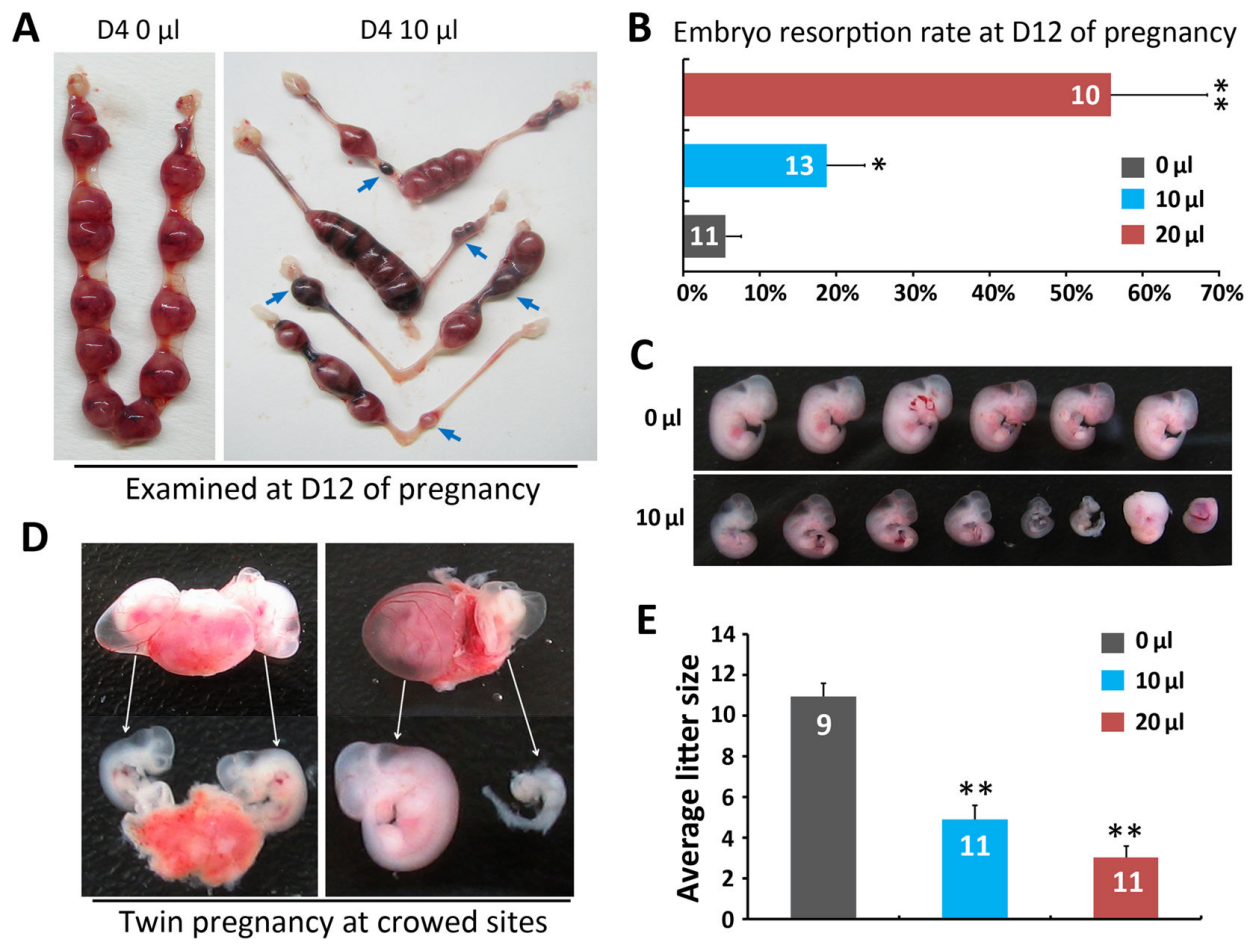
Abnormal timing and spacing of embryo implantation after intrauterine fluid infusion caused pregnancy loss at mid-gestation. (**A**) Demonstrative photos of Day12 uteri after 0 μl and 10 μl intrauterine fluid infusion on Day4 morning. Red arrows showed abnormal implantation sites (retarded growth/crowded site). (**B**) Embryo resorption rate at Day12 after 0 μl, 10 μl and 20 μl intrauterine fluid infusion on Day4 morning (*P < 0.05, **P < 0.01) *t* test, Error bars represent S.E.M ). (**C**) Representative photos of normal embryo and retarded/resorbed embryos from 0μl and 10 μl groups at Day12. (**D**) Examples of twin pregnancy discovered at crowed implantation sites on Day12, note that two embryos were grown on one placenta, in the right panel, one of the twin embryo showed severely retarded growth and tended to be resorbed. (**E**) Average litter size at birth (**P < 0.01) *t* test, error bars represent S.E.M. Numbers within bars indicate the number of mice examined.

To examine the ongoing effects of intrauterine fluid infusion at preimplantation, we examined the pregnancy status at mid-term (Day 12) in 0 μl, 10 μl and 20 μl group. As shown in Figure 3A,B, while mice from 0 μl group mostly showed well developed implantation sites, the 10 μl group showed significantly increased embryo resorption rates and retarded embryo growth. These adverse effects were more pronounced in 20 μl group (Figure 3B). In addition,we usually found twin embryos grown on one placenta from the crowded implantation sites and some of them showed significantly retarded growth and tends to be resorbed (Figure 3D). At the end of pregnancy, the litter size was significantly decreased in both 10 μl and 20 μl group as compared with the 0 μl group (Figure 3E). 

### Intrauterine infusion of saline at preimplantation changed epithelium markers for embryo implantation

**Figure 4:**
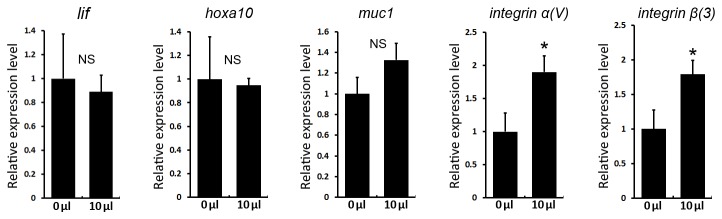
Uterine gene expression after 0μl and 10 μl fluid infusion. RNA was prepared from Day4 uteri (16:00) after 0 μl or 10 μl fluid infusion in the morning (08:30), and were processed for real-time quantitative PCR analysis using gene-specific primers of *Lif, Hoxa10, Muc1, Integrin α*(*v*) *and* *Integrin* β(3). n=5 for each gene. (NS: P > 0.05; *P < 0.05). Error bars represent S.E.M, *t* test.

To further explore the potential effects of intrauterine fluid infusion on uterine gene expression, we examined gene expression profiles relating to uterine receptivity including *Lif, Hoxa10, Integrin α*(*v*) *and Integrin* β(3) [23–27], because these molecules have shown changed expression in the endometrium of hydrosalpinx patients. We also examined *Muc1*, a barrier glycoprotein expressed at apical surface of uterine epithelial lining [28] and has been proposed as a potential marker to be distinguished for hydrosalpinx [29]. As shown in Figure 4, we found that compared with 0 μl group, only *integrin* α(*v*) *and integrin* β(3) showed significant upregulation after 10 μl fluid infusion. Since the intrauterine infusion was performed on the morning of Day4 and the uterine gene expression were examined in the afternoon of the same day, such unchanged expression of uterine receptivity markers was not surprising to us, while the upregulated expression of attachment reaction related molecules (*lntegrin α(v*)* β(3*)) might suggest a compensatory reaction in confront of the excessive intrauterine fluid. 

## Discussion

Our present study revealed that excessive uterine fluid at preimplantation, even non-toxic as saline, could cause abnormal embryo implantation thus consequent embryo loss. The adverse effects of excessive uterine fluid are not due to a direct “flush out” of embryos, but mainly caused a delayed timing and the abnormal implantation location. These results reinforced the previously established concept that a shot delay or/and abnormal location of embryo implantation, by any means (genetic deletion, pharmacological treatment), will trigger ripple adverse effects for ongoing pregnancy in both rodents and human [20,30–32]. Most importantly, the adverse pregnancy outcome observed in present study is strikingly similar to the decreased implantation rate and increased miscarriage rate in patients with hydrosalpinx undergoing IVF-ET cycle, therefore provided a pathophysiologically related animal model in explaining the causes of hydrosalpinx’s adverse effects in implantation and ongoing pregnancy.

From our observation, the most possible effect of the excessive intrauterine fluid by the time of embryo implantation would be a direct interference of embryo-uterine apposition, making the embryos floating in the uterine lumen beyond the expected timing of implantation initiation. Previous studies on the timing of embryo spacing have shown that by the time of Day4 10:00 am, the process of embryo spacing have not been initiated for >70% of embryos, while the spacing process become gradually perfected until Day4 24:00 pm [33]. Such timing of embryo spacing has also been similarly observed in more recent publication [34]. In our experimental system, the infusion of excessive fluids in the uterine lumen at the Day4 08:30 (before the embryo spacing process begins) would mostly cause the embryos “floating” in the lumen thus make it difficult to be accurately transported. In human, it has been suggested that the transferred embryos could “float off” the transfer location and do not implant at the transferred site [35,36], possibly due to the excessive fluid. Also, the excessive luminal fluid as a physical stimulus might also trigger abnormal uterine peristalsis, which would be an important cause for aberrant embryo spacing [18].

Previous studies in both rodent and human studies have demonstrated that once the “on time” and “on site” implantation were disrupted, it will lead to compromised embryo development and pregnancy loss at mid-gestation [20,30–32]. Our data recapitulated this concept and suggested that the abnormal timing and location of embryo implantation might be a common cause for the increased miscarriage rates in patients with hydrosalpinx undergoing IVF-ET cycle. Another interesting discovery in this study is the resorbed twin embryo at the crowded implantation sites, such observation is similar to the clinical situation of “vanishing twin” syndrome, which refer to the phenomena when one of twin/multiple fetuses disappears in the uterus due to retarded growth/miscarriage of one twin [37,38]. The surviving twin fetus have been reported to show increased risks for postnatal abnormalities [38]. 

Besides the mechanistic aspects, it has been reported that the hydrosalpinx also influenced the markers of endometrial receptivity. Previous studies have shown that in the presence of hydrosalpinges, the expression of *Lif, Hoxa10* and *Integrin* α(*v*)β(3) is signiﬁcantly reduced in the window of implantation compared with normal controls, and the expression could be restored by removal of hydrosalpinges [23–27]. These results suggested that the leakage of hydrosalpinx fluid into the uterine cavity might change the uterine gene expression for implantation. In our current model, the infusion of non-toxic fluid (saline) didn’t trigger similar changes regarding the gene expression (Figure 4) as in hydrosalpinx patients, this result, however, was not totally unexpected, because compared with the chronic leakage of hydrosalpinx fluid in patients, our model only provide a transient exposure of excessive intraluminal fluid. Also, it implied that the changed uterine receptivity in hydrosalpinx patients might be due to the inflammatory /toxic factors of the hydrosalpinx fluid. Unexpectedly, in our intrauterine infusion model, we observed an increased expression of *lntegrin* α(*v*) *and* β(3), suggesting that when the uteri encountered unfavorable excessive fluid environment, it triggers a compensatory effect that express more “adhesive” molecules such as *Integrinα*(*v*) *and* β(3) as to increase to chance to catch the floating embryos. In addition to the changed uterine receptivity, the excessive luminal fluid might also dilute important soluble factors (cytokines, chemokines et.al) within the uterine lumen, which might decrease the efficiency of implantation. 

Once exogenous fluid enters into uterine lumen, the ion pump and aquaporins might also respond to excessive fluid. We have also examined the expression of important uterine ion channel (*CFTR*) and water channel (*Aqp5*) according to previously reports [1,39]. We observed an interesting result that after intrauterine fluid infusion, the uterine chloride channel *CFTR*, is upregulated, while the major uterine water channel *Aqp5* showed no obvious change (Fig. S2). The upregulation of *CFTR* upon excessive uterine fluid infusion might be caused by physical stimulus (such as epithelium stretch) triggered by excessive intrauterine fluids, and the elevated *CFTR* is supposed to enhance the secretion of intrauterine fluids, further exaggerating the excessive intrauterine fluids. This data is in concert with previous report that *CFTR* was indeed involved in the pathogenesis of hydrosalpinx [40]. 

Finally, besides the implications for hydrosalpinx, the present study also raised attentions for the basic study of embryo implantation. Since intrauterine luminal injection of drugs/siRNAs/antibodies is one of the methods to study implantation related factors *in vivo*, our present data raised serious caution for this methods because the vehicle (saline) alone, if excessive, would efficiently affect implantation rate and ongoing pregnancy, which have also been noticed and mentioned by other researchers [41]. 

## Supporting Information

Figure S1 **Luminal closure process was delayed after intrauterine saline infusion.** (A) luminal closure status at 16:00 Day4 pregnancy. The black arrow showed unclosed lumen, the white arrow showed almost completed luminal closure. (B) Luminal closure status at 24:00 Day4 pregnancy. The black arrow showed unclosed lumen, the white arrow showed completed luminal closure. LE: luminal epithelia; GE: glandular epithelia; BL: blastocyst.
(PDF)

Figure S2 **Uterine gene expression of Aqp5 and CFTR after 0μl or 10μl fluid infusion.** RNA was prepared from Day4 uteri (16:00) after 0μl or 10μl fluid infusion in the morning (08:30), and were processed for real-time RT-PCR analysis numbers in the bars represents number of mice used for each group. (NS: P > 0.05; *P < 0.05). Error bars represent S.E.M, t test.
(PDF)
