# Supplementary figures and images for: Excessive Intrauterine Fluid Cause Aberrant Implantation and Pregnancy Outcome in Mice
Source: PLoS One. 2013 Oct 23;8(10):e78446. doi: 10.1371/journal.pone.0078446 (PMC3806840; doi:10.1371/journal.pone.0078446)

**A****Day4 16:00****0 $\mu$ l**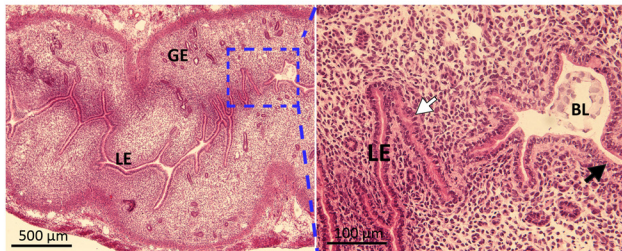**10 $\mu$ l**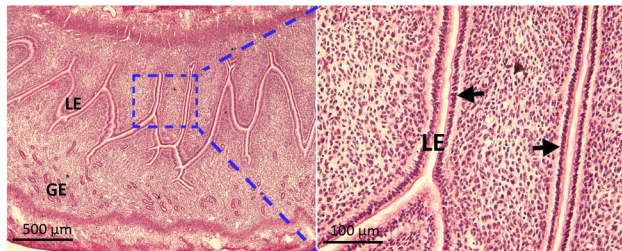**B****Day4 24:00**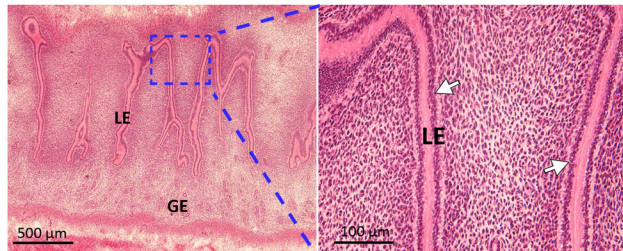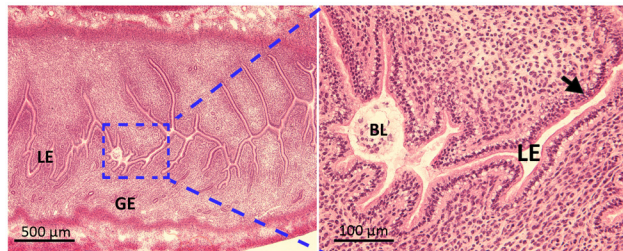

Supplement: Figure S1 — Luminal closure process was delayed after intrauterine saline infusion. (A) luminal closure status at 16:00 Day4 pregnancy. The black arrow showed unclosed lumen, the white arrow showed almost completed luminal closure. (B) Luminal closure status at 24:00 Day4 pregnancy. The black arrow showed unclosed lumen, the white arrow showed completed luminal closure. LE: luminal epithelia; GE: glandular epithelia; BL: blastocyst. (PDF) [file pone.0078446.s001.pdf]

*Aqp5*

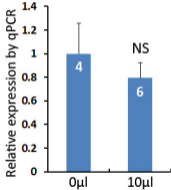

*CFTR*

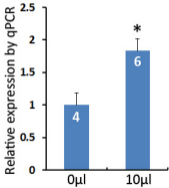

Supplement: Figure S2 — Uterine gene expression of Aqp5 and CFTR after 0μl or 10μl fluid infusion. RNA was prepared from Day4 uteri (16:00) after 0μl or 10μl fluid infusion in the morning (08:30), and were processed for real-time RT-PCR analysis numbers in the bars represents number of mice used for each group. (NS: P > 0.05; *P < 0.05). Error bars represent S.E.M, t test. (PDF) [file pone.0078446.s002.pdf]
